# NLOS/LOS identification with LightGBM ensemble

**DOI:** 10.1371/journal.pone.0353288

**Published:** 2026-07-10

**Authors:** Xiaofeng Yang, Zhao Wu, Danlei Mo

**Affiliations:** School of Physics and Telecommunication Engineering, Yulin Normal University, Yulin Guangxi, China; Hohai University, CHINA

## Abstract

Non-Line-of-Sight (NLOS)/Line-of-Sight (LOS) identification is crucial to accurate Ultra-Wideband (UWB) positioning. The current Machine Learning solutions to this problem have either too many parameters to tune or too simple features to input, which lead to unsatisfactory performance. To address this issue, this paper proposed a novel binary classifier called LightGBM Ensemble which integrates multiple LightGBMs in parallel with multi-scale patch extraction. The heterogeneous LightGBM ensemble architecture boosts the prediction power of individuals. The multi-scale patch extraction scheme extracts informative features from time-frequency domains. Extensive experiments on an open-source dataset were conducted to evaluate the proposed approach, which proves its superior classification performance and generalization performance with feasible complexity compared to the state-of-the-art Deep Learning and Decision Trees methods.

## 1. Introduction

Ultra-Wideband (UWB) technology plays an important role in indoor positioning, for the sake of its fine time resolution and high positioning accuracy in general [1]. However, its positioning performance degrades dramatically in Non-Line-of-Sight (NLOS) scenarios where blocked or scattered signals travel longer paths than Line-of-Sight (LOS) propagation and introduce bias to ranging [2]. Therefore, NLOS/LOS identification is crucial to UWB positioning performance enhancement [3]. Recent literature in UWB NLOS/LOS identification concentrate on various Machine Learning (ML) methods which tackle this issue as binary classification tasks. As for the branch of Deep Learning (DL), Reference [4] proposed a Convolutional Neural Network- Bidirectional Long Short-Term Memory (CNN-BiLSTM) method to jointly learn UWB Channel Impulse Response (CIR) temporal features and Voronoi-based spatial propagation features for NLOS/LOS identification. Paper [5] introduced bidirectional encoder representations from transformers with fuzzy probabilities (F-BERT) that comprised both the time and energy characteristics of NLOS/LOS CIR sequences. Article [6] applied Gramian angular field (GAF) encoding to CNN model to capture the temporal correlations within NLOS/LOS signals. Authors in [7] designed a domain-adversarial neural network to perform adversarial learning of domain-invariant NLOS/LOS CIR features. Aforementioned DL architectures contain many parameters to tune and require large amount of data to train which are computational complex and time-consuming. As for the branch of Decision Trees, the work in [8] used fuzzy-logic-enhanced Light Gradient Boosting Machine (LightGBM) for reliable NLOS/LOS identification. The study in [9] implemented robust NLOS/LOS classification based on Extreme Gradient Boosting (XGBoost) with feature selection using the Pearson Correlation Coefficient. Aforementioned Decision Trees approaches can only handle limited 1-D sequential features which lead to unsatisfactory NLOS/LOS identification performance.

This paper propounded a novel UWB NLOS/LOS identification strategy based on LightGBM ensemble with multi-scale patch extraction. The LightGBM ensemble integrates multiple LightGBM classifiers in parallel to form a more powerful classification “committee” than single LightGBM. On the other hand, multi-scale patch extraction from the raw data generates 2-D feature subsamples which contains distinguishable characteristics of NLOS/LOS signals in both time domain and frequency domain as input to the LightGBM ensemble, achieving higher NLOS/LOS identification accuracy than 1-D sequential features. Evaluation of this strategy with open-source dataset proves its superior NLOS/LOS identification performance. The following lists the main innovation and contributions of this paper:

(1) This paper designed a competitive binary classification framework with LightGBM ensemble which combines the merits of both DL and Decision Trees, i.e., high classification accuracy and feasible architecture. More importantly, this paper put forth a novel NLOS/LOS identification methodology based on the proposed LightGBM ensemble model.
(2) For the first time, multi-scale patch extraction was applied to UWB CIR signal to capture effective time-frequency domain features in 2-D images to discriminate NLOS/LOS signals.
(3) Substantial evaluation experiments were conducted to prove the advantage of the proposed scheme comparing with the up-to-date DL technique CNN-BiLSTM and the Decision Trees counterpart single LightGBM.

## 2. Methods

### 2.1. LightGBM

Gradient Boosting Decision Trees (GBDT) suggests a robust gradient descent “boosting” paradigm for additive decision trees to execute either regression or classification tasks [10]. The main defeat of GBDT is that for each feature, it needs to go through all the data for information gain calculation with low efficiency and poor scalability for big data and numerous features.

To address this issue, LightGBM speeds up GBDT by about 20 times via two techniques: Gradient-based One-Side Sampling (GOSS) and Exclusive Feature Bundling (EFB) [11]. GOSS excludes the data with small gradients, i.e., less information gain, which effectively reduces the data size without degrading the calculation accuracy by much. On the other hand, EFB bundles mutually exclusive features with rarely simultaneous non zero values, which effectively reduces the feature dimension without hurting the tree splitting accuracy by much.

GOSS first sorts the absolute gradient value $g_{i}$ of original training data $T=\overset{I}{\underset{\text{1}}{\left\{X_{i},\;y_{i}\right\}}}$ in descending order, and keeps the top a×|T| training data to form a subset *A* with the larger gradients, then randomly samples b×|T\A| training data to form a subset *B* with the smaller gradients, where *a* and *b* are the sampling ratio of the large and small gradient data respectively. At last, split the final training data subset S=A∪B for *j*-th feature $X_{ij}$ at value $v_{j}$ to maximize the information gain in Eq (1).


$$\begin{array}{c} Gain\left(v_{j}\right)=\frac{{\left(\sum_{i=\text{1}}^{\left|A_{l}\right|}g_{i}+\frac{\text{1}-a}{b}\sum_{i=\text{1}}^{\left|B_{l}\right|}g_{i}\right)}^{\text{2}}}{\left|A_{l}\right|+\left|B_{l}\right|}+\frac{\text{1}}{\left|S\right|}\cdot \frac{{\left(\sum_{i=\text{1}}^{\left|A_{r}\right|}g_{i}+\frac{\text{1}-a}{b}\sum_{i=\text{1}}^{\left|B_{r}\right|}g_{i}\right)}^{\text{2}}}{\left|A_{r}\right|+\left|B_{r}\right|} \end{array}$$ (1)

Where $A_{l}=\left\{X_{ij}\in A\;and\;X_{ij}\leq v_{j}\;\right\}$, $A_{r}=\left\{X_{ij}\in A\;and\;X_{ij}\geq v_{j}\;\right\}$, $B_{l}=\left\{X_{ij}\in B\;and\;X_{ij}\leq v_{j}\;\right\}$, $B_{r}=\left\{X_{ij}\in B\;and\;X_{ij}\geq v_{j}\;\right\}$ and the factor $\frac{\text{1}-a}{b}$ normalized the sum of gradients over *B* back to T\A.

EFB first ranks the features by the count of nonzero values in descending order. If the count of some feature is larger than the maximum conflict count, then create a new bundle; otherwise, add the feature to an existing bundle with a small maximal conflict rate gamma in each bundle. Then combine the features in the same bundle by adding guard offset to the original feature value before merging such that each feature can be identified in the merged bundle.

### 2.2. LightGBM Ensemble

This paper proposed to integrate multiple LightGBM classifiers in parallel as a powerful “committee” to mitigate the classification bias of individual classifier and boost the generalization performance of the ensemble model.

As for binary classification, the goal is to construct an ensemble model of *M* LightGBMs, each with *N* decision trees, for accurate estimation of a function f(X) that maps input X to a binary label y∈{1,−1} with minimum value of the loss function l(y,f(X)). While the decision trees within each single LightGBM are addictive, the features extracted from input X for different LightGBMs are different, therefore this is a heterogeneous ensemble of LightGBMs based on different feature views. LightGBM ensemble average the predicted probabilities of each class from each LightGBM in parallel and label the input data with the maximal probability class. **Algorithm 1** states the binary classification method with the novel LightGBM ensemble.

**Algorithm 1.** Binary Classification with LightGBM Ensemble

Input $X=\overset{M}{\underset{m=\text{1}}{\left\{X_{m}\right\}}}$; *% Input feature* $X_{m}$ *to m-th LightGBM.*

Output label y∈{1,−1};

For *m = 1:M*

  Training data $T_{m}=\overset{I}{\underset{i=\text{1}}{\left\{X_{mi},\;y_{i}\right\}}}$;

  GOSS($T_{m}$);

  EFB($T_{m}$);

  $f_{m\text{0}}\left(X_{m}\right)=\text{0}$;

  For *n = 1:N % Construct each LightGBM with N decision trees.*

  Gradient $g_{i}=\frac{\text{2}y_{i}}{\text{1}+exp\left(\text{2}y_{i}f_{m(n-\text{1})}\left(X_{mi}\right)\right)}$;

  Construct *J*-terminal node tree $\overset{J}{\underset{\text{1}}{\left\{R_{jn}\right\}}}$with pseudo training data $\overset{I}{\underset{i=\text{1}}{\left\{X_{mi},\;g_{i}\right\}}}$ to maximize the information gain in Eq.(1);

  Scaling factor $\sigma_{jn}=\frac{\sum_{X_{mi}\in R_{jn}}g_{i}}{\sum_{X_{mi}\in R_{jn}}\left|g_{i}\right|\left(\text{2}-\left|g_{i}\right|\right)}$;

  $f_{mn}\left(X_{m}\right)=f_{m(n-\text{1})}\left(X_{m}\right)+\sum_{j=\text{1}}^{J}\sigma_{jn}{\text{1}}_{X_{m}\in R_{jn}}$;

  End For

  $P_{m}\left(\text{y}=\text{1}|X_{m}\right)=\frac{\text{1}}{\text{1}+\text{exp}(-\text{2}f_{mn}\left(X_{m}\right))}$;

  $P_{m}\left(\text{y}=-\text{1}|X_{m}\right)=\frac{\text{1}}{\text{1}+\text{exp}(\text{2}f_{mn}\left(X_{m}\right))}$;

End For

  *% Ensemble prediction of M LightGBMs:*

  $P\left(\text{y}=\text{1}|X\right)=\frac{\sum_{m=\text{1}}^{M}P_{m}\left(\text{y}=\text{1}|X_{m}\right)}{M}$;

  $P\left(\text{y}=-\text{1}|X\right)=\frac{\sum_{m=\text{1}}^{M}P_{m}\left(\text{y}=-\text{1}|X_{m}\right)}{M}$;

  $\hat{y}=2\times 1_{\mathbb{P}(\text{y}=1|\text{X}})>\mathbb{P}(\text{y}=-1|\text{X}\})\}-1$

End Algorithm

### 2.3. Multi-scale patch extraction

Theoretically, better classification accuracy can be achieved with more features from various domains. Morlet Wavelet Transform (MWT) outputs wavelet power spectrums which are then normalized into grey scale images as the feature input, incorporating feature information from both time domain and frequency domain that will enhance the classification accuracy of using only time domain features [12].

**Fig 1:**
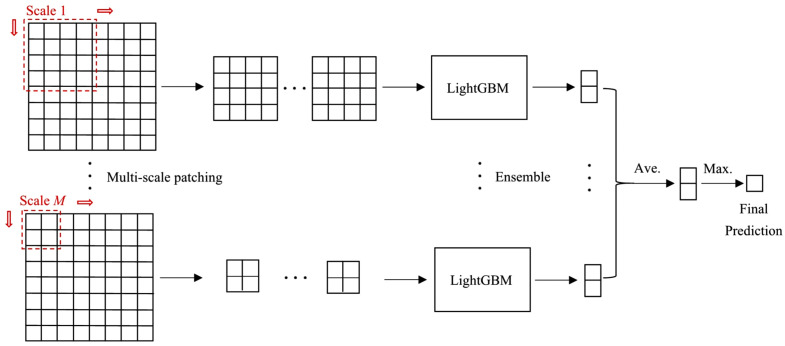
The proposed approach.

This paper applied multi-scale sliding panels to resample the raw feature images from MWT before input to the classifier, as illustrated in Fig 1. Although each subsample contains less information than the raw image, massive multi-scale subsamples as numerous local snapshots of the raw data completely dig out both fine-grained characteristics and long-term trends among high dimensional data, which result in better representation learning of the contextual information, therefore achieve better classification performance when training with them than with the raw feature image [13] [14].

### 2.4. The proposed NLOS/LOS Identification Approach

This paper proposed the following NLOS/LOS identification method based on Light Ensemble with multi-scale patch extraction.

#### Preparing training inputs.

Step 1: Perform MWT on UWB CIR data to get wavelet power spectrums then normalize them into d×d grey scale images.

Step 2: Perform *M*-scale patch extraction on the raw images with multiple sliding panel dimension $\left\lfloor d/{\text{2}}^{n}\right\rfloor \times \left\lfloor d/{\text{2}}^{n}\right\rfloor$ and sliding step size *s* to get multiple subsamples as training inputs.

#### Learning LightGBM Ensemble model.

Input *M*-scale subsamples to train *M* LightGBMs in LightGBM Ensemble respectively, as **Algorithm 1**.

#### Predicting the NLOS/LOS label.

Average the predicted probabilities of each class from each LightGBM and label the input data with the maximal probability class.

## 3. Results and discussion

The proposed approach was implemented on the open-source PyTorch platform with the primary parameters setup listed in Table 1. The evaluation experiments were conducted on a Dell laptop with Intel Core 7 CPU and 16G RAM with open-source dataset UPT [15]. The dataset includes UWB CIR measurements from 4 different indoor environments: residential apartment, small apartment, industrial workshop and office. 500 LOS CIR samples and 500 NLOS CIR samples were randomly selected from each environment to obtain 4000 CIR samples in total for this study. 4-fold cross-validation was applied to prevent overfitting. Please note that cross-validation splitting is performed at the original CIR sample level before any resampling, ensuring that all subsamples from the same original CIR sample remain strictly within the same fold Table 1.

**Table 1:** Primary parameters setup.

Parameters	Value
Sampling ratio of large gradient data	*a* = 0.1
Sampling ratio of small gradient data	*b* = 0.1
Maximal conflict rate in each feature bundle	γ=0
Learning rate	λ=0.01
Number of LightGBM	*M* = 3
Number of iterations for each LightGBM	*N = 100*
Sliding panel step size	*s* = 1
Sliding panel dimension for d×d image	⌊d/16⌋×⌊d/16⌋, ⌊d/8⌋×⌊d/8⌋, ⌊d/4⌋×⌊d/4⌋.

NLOS CIR samples were labeled as positive class and LOS CIR samples were labeled as negative class. There are 4 types of possible outputs: True Positive (TP) and True Negative (TN) denote NLOS and LOS CIR samples were correctly classified respectively, while False Positive (FP) and Fauls Negative (FN) denote LOS and NLOS CIR samples were incorrectly classified respectively. The metrics for classification performance evaluation are accuracy, precision, recall and F1-score. The calculation formulations of each metric are as Eq. (2)-(5). The accuracy metric indicates the correctly labeled ratio among all the data, which is a general indicator of classification performance. The precision metric indicates the ratio of true positive data among all the labeled positive data, which should be emphasized more when the cost of FP is more serious. The recall metric indicates the correctly labeled ratio among all the true positive data, which should be emphasized more when the cost of FN is more serious. The F1-score metric is a composite indicator of precision and recall, which should be emphasized more when the impact of both FP and FN is equally important. In the case of positioning application, correctly identify both NLOS and LOS signal are considered to be equally important, therefore this paper emphasizes more on the accuracy and F1-score metrics.


$$\begin{array}{c} \text{Accuracy}=\frac{TP+TN}{TP+TN+FP+FN} \end{array}$$ (2)

$$\begin{array}{c} \text{Precision}=\frac{TP}{TP+FP} \end{array}$$ (3)

$$\begin{array}{c} \text{Recall}=\frac{TP}{TP+FN} \end{array}$$ (4)

$$\begin{array}{c} F1-score=\frac{\text{2}\times Precision\times Recall}{Precision+Recall} \end{array}$$ (5)

The first experiment was to compare the classification performance of the proposed method LightGBM Ensemble with the up-to-date DL technique CNN-BiLSTM [4] and the Decision Trees counterpart single LightGBM [8]. Please note that for fair comparison, the input to CNN-BiLSTM are the same grey scale wavelet images applied in LightGBM Ensemble. The results are listed in Table 2. LightGBM Ensemble achieves 0.97 accuracy and F1-score with slight standard deviation (std) derived from the 4-fold cross-validation, and maximal P-value of 0.0006 and 0.0003 comparing with CNN-BiLSTM and Single LightGBM respectively derived from paired t-test on the fold-level results of above metrics. P-value < 0.05 indicates that at 0.05 significance level, the classification performance improved by the proposed approach in comparison to each baseline is statistically significant therefore demonstrates that the proposed approach is superior and reliable. This stems from the boosting LightGBM “committee”, robust 2-D feature extraction from time-frequency domain and contextual information mining with the multi-scale patch extraction scheme. On the other hand, the classification performance of CNN-BiLSTM as a DL model degrades on such a relatively small training dataset and the classification performance of Single LightGBM is limited by relatively simple architecture and1-D sequential features Table 2.

**Table 2:** Classification performance comparison.

Methods	Accuracy/std	Precision/std	Recall/std	F1-Score/std	P_max_
CNN-BiLSTM	0.92/0.01	0.93/0.02	0.91/0.02	0.92/0.02	0.0006
Single LightGBM	0.92/0.01	0.93/0.02	0.90/0.02	0.91/0.02	0.0003
LightGBM Ensemble	0.97/0.01	0.98/0.01	0.97/0.01	0.97/0.01	NA

**Fig 2:**
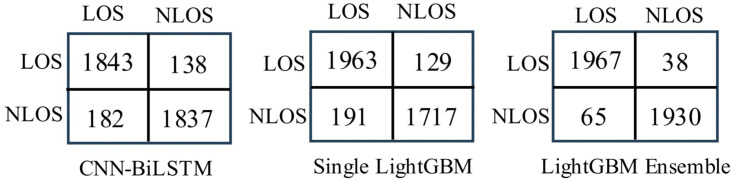
Confusion matrix.

Fig 2 depicts the confusion matrix of the three binary classification methods, the rows of which represents the true labels and the columns of which represents the predicted labels. The confusion matrix intuitively shows that the number of TP and TN samples of LightGBM Ensemble is the largest and the number of FP and FN samples of LightGBM Ensemble is the smallest among the three binary classifiers in comparison which confirms the solid classification performance of LightGBM Ensemble, for the sake of more powerful heterogeneous LightGBM ensemble architecture and more informative feature representation learning.

The second experiment was to evaluate the generalization performance of the proposed strategy LightGBM Ensemble in various environments. The classifier was trained with the samples from three of the four environments and tested with the samples from the remaining environment. The outcome is listed in Table 3. Testing with the environments of residential apartment, small apartment and office obtain larger than 0.90 accuracy and F1-score, which achieve comparable classification performance in the three types of environments. While testing with the environment of industrial workshop, the classification performance degraded slightly, which can be attributed to the dense NLOS propagation characteristics of industrial workshop quite different from the other three scenarios, i.e., industrial workshop is deployed with massive metallic machinery, resulting in highly reflective surfaces that cause more severe multipath propagation and signal distortion than the other three scenarios. In general, LightGBM Ensemble achieves satisfactory generalization performance Table 3.

**Table 3:** Generalization performance.

Testing Environments	Accuracy/std	Precision/std	Recall/std	F1-Score/std
Residential Apartment	0.91/0.01	0.92/0.01	0.91/0.01	0.91/0.01
Small Apartment	0.92/0.01	0.92/0.01	0.92/0.01	0.92/0.01
Industrial Workshop	0.86/0.01	0.84/0.01	0.86/0.01	0.85/0.01
Office	0.90/0.01	0.91/0.01	0.89/0.01	0.90/0.01

The third experiment was to perform ablation analysis for the proposed model LightGBM Ensemble which integrated two critical parts, i.e., the heterogeneous LightGBM ensemble architecture and multi-scale patch extraction (MSPE) scheme. Table 4 compares the classification performance of the proposed model to that of its counterparts without these two critical parts respectively. As shown in Table 4, LightGBM Ensemble without MSR improves by 0.02 accuracy and 0.03 F1-score compared to Single LightGBM which demonstrates the effectiveness of the heterogeneous LightGBM ensemble architecture. LightGBM Ensemble with MSR improves by 0.03 accuracy and F1-score compared to LightGBM Ensemble without MSR which proves the effectiveness of the MSR scheme Table 4.

**Table 4:** Ablation analysis.

Methods	Accuracy/std	Precision/std	Recall/std	F1-Score/std
Single LightGBM	0.92/0.01	0.93/0.02	0.90/0.02	0.91/0.02
LightGBM Ensemble w/o MSPE	0.94/0.01	0.95/0.01	0.93/0.01	0.94/0.01
LightGBM Ensemble w MSPE	0.97/0.01	0.98/0.01	0.97/0.01	0.97/0.01

At last, the time complexity of the three methods is compared in Table 5. The time complexity of CNN is $O\left(IWHL+ISU^{\text{2}}\right)$ [5], where *I* is the number of training data, *W* and *H* is the width and height dimension of the input image, *L* is the number of CNN convolutional layers, *S* is the input sequence length, *U* is the number of BiLSTM hidden units. The time complexity of Single LightGBM is O(ISB) [11], where *B* is the number of feature bundles. The time complexity of LightGBM Ensemble is O(IWHB), which scales with the number of training data, the input dimension and the number of feature bundles but not with the number of LightGBMs as multiple LightGBMs are integrated in parallel and execute classification at the same time. It is obvious that CNN-BiLSTM is the most complex and Single LightGBM is the most efficient, while the proposed LightGBM Ensemble achieves the best NLOS/LOS identification performance with feasible complexity.

**Table 5:** Time complexity comparison.

Scheme	Complexity
CNN-BiLSTM	$O\left(IWHL+ISU^{\text{2}}\right)$
Single LightGBM	O(ISB)
LightGBM Ensemble	O(IWHB)

Note: *I* is the number of training data, *W* and *H* is the width and height dimension of the input image, *L* is the number of CNN convolutional layers, *S* is the input sequence length, *U* is the number of BiLSTM hidden units and *B* is the number of LightGBM feature bundles. For LightGBM Ensemble, its time complexity O(IWHB) also reflects per-model computational cost, the total computational cost scales linearly with the number of LightGBM models *M*, but as they are independent, training can be perfectly parallelized in practice and the execution time counts once regardless of *M*.

## 4. Conclusions

This paper provided a “boosting” paradigm of LightGBM Ensemble with multi-scale patch extracted features in time-frequency domain, which was successfully applied to NLOS/LOS identification as a binary classification task and outperforms the up-to-date DL model CNN-BiLSTM and the Decision Trees counterpart single LightGBM with feasible complexity. The future work will extend the proposed model to multi-class classification applications.
